# USER EXPERIENCES OF OLDER ADULTS NAVIGATING AN ONLINE DATABASE OF COMMUNITY-BASED PHYSICAL ACTIVITY PROGRAMS

**DOI:** 10.1093/geroni/igad104.1986

**Published:** 2023-12-21

**Authors:** Denise Connelly, Ashley Lowndes

**Affiliations:** Western University, London, Ontario, Canada; University of Western Ontario, London, Ontario, Canada

## Abstract

As cataloguing health care services becomes more internet-based, older adults are increasingly expected to navigate services online. Previous studies have outlined the needs, barriers, and facilitators of older adults using eHealth, however, none included user experiences in navigating websites. Connecting community-living older adults with local, high quality, community-based physical activity programs is a gap in efforts to promote physical activity by older adults. This study aimed to: 1) explore older adult user experiences navigating an online health database of local physical activity programs; 2) compare navigational feedback with age-friendly website design guidelines; and 3) assess online database completeness. Focus groups, including guided navigation tasks and a semi-structured interview script, gathered user experiences of fifteen older adults (≥65). A literature review produced three age-friendly best practice website design guidelines. A website search for local physical activity programs was completed. The design of the online database website was challenging for older adult participants to navigate and was not ’intuitive’. In navigating the online database, the older adults identified multiple discrepancies with established guidelines for designing age-friendly websites. A total of 187 local physical activity programs were missing from the database. Findings provide novel insight into user experiences of older adults navigating online health and physical activity program websites. Redesign following age-friendly website recommendations would empower older adults in use of online databases and promote awareness of local physical activity programs. Health care providers need reliable and age-friendly online resources to link their patients with local physical activity programs to promote healthy aging.

